# Consumer Behavior in the Indian Online Eyewear Market After COVID-19: A Trend or Public Concern?

**DOI:** 10.7759/cureus.77416

**Published:** 2025-01-14

**Authors:** Sweatha Santhababu, Samuel Livingstone Kumaran, Tamilselvan Pachiyappan

**Affiliations:** 1 Department of Optometry, Panimalar College of Allied Health Sciences, Chennai, IND; 2 Department of Optometry, Faculty of Allied of Health Sciences, Sri Ramachandra Institute of Higher Education and Research, Chennai, IND; 3 Department of Optometry, Mangala College of Allied Health Sciences, Mangalore, IND

**Keywords:** consumer behavior, covid-19 impact, eyewear buying, indian e-commerce, online eyewear market

## Abstract

Background: The growing trend of online shopping has significantly transformed the global eyewear market since the onset of COVID-19. However, concerns about the quality and fit of spectacles purchased online persist. This study aims to understand consumer satisfaction and preferences related to online spectacle purchases in India.

Methods: A cross-sectional online survey was conducted with participants who had purchased eyewear through e-commerce platforms. The survey collected demographic data, information on refractive errors, frame and lens preferences, and user experiences related to appearance, fit, comfort, safety, vision, and symptoms associated with spectacles purchased online.

Results: Among 85 respondents, the majority who opted for single-vision spectacles (66, 78%) were young adults, with myopia (55, 65%) being the most common refractive error. Participants predominantly favored full-rim frames (56, 66%), plastic frame material (49, 58%), and blue-light control lenses (58, 68%). While 58 (68%) expressed satisfaction with the appearance and 56 (66%) with the comfort of their spectacles, 27 (32%) reported safety concerns due to symptoms such as headaches (17, 20%) and blurred vision (14, 17%). A comparison between the questions "Which method of purchasing spectacles do you often prefer?" and "Based on your recent experience, where do you think you will purchase your next pair of spectacles?" showed no significant difference (p = 0.428).

Conclusion: This study highlights consumer trends in online spectacle purchases among the Indian population after COVID-19. A significant portion of the younger population, primarily single-vision lens users, reported satisfaction with their spectacles. However, concerns regarding fit, comfort, and safety emphasize the need for professional oversight and quality assurance, which are more readily achievable with offline purchases. Individuals who often prefer online purchases remain inclined to continue purchasing spectacles online.

## Introduction

Purchasing spectacles online has become more common than purchasing from private optometry practices or in optical stores in developed countries [1]. Global sales of commodities purchased online have risen significantly in recent years following the onset of COVID-19 [2,3]. Online purchases of prescription spectacles account for approximately 6% of the market in the United Kingdom, have surpassed 20% in the United States, and continue to grow, while India is expected to reach double digits soon [4-6]. It is also reported that the e-commerce volume growth for eyewear and accessories has increased by 47% since 2019, when the COVID-19 pandemic started [3,7]. Online eyewear purchases are promoted through a direct-to-consumer model, targeting young people with stylish spectacle frames and sunglasses [5]. It is definite that eye care practitioners are concerned about the purchase of eyewear through e-commerce platforms whereas the general public feels the online purchase to be affordable, fair, and accessible to several brands [5,6,8]. In the current era, e-commerce’s steep rise has shifted buyers from traditional stores to Internet buying across various products [9]. An online survey conducted in India revealed that distinctive offers, a wide range of collections, and convenience were the top reasons for people preferring online purchases of eye care products [10]. The convenience of having a product booked within minutes, home delivery, cash on delivery, and dedicated web pages with face recognition models for the selection of frames are added attractions. The 2020 Internet Influence Report by the Vision Council stated that approximately 24% of American adults purchased prescription eyeglasses online, a trend primarily driven by the COVID-19 pandemic and is expected to grow in the coming years [11].

Online purchases bring responsibility to the buyers to enter their correct facial and frame measurements, prescription details and other specifications to fulfill their spectacle needs. Buyers can order single-vision glasses, bifocals, progressive additive lenses (PALs), ready-made reading glasses, sunglasses, prescription single-vision powered sunglasses, and much more from their preferred sites. Ordering prescription eyeglasses online brings a risk factor, as the dispensed spectacles are directly delivered to the patient without any standard spectacle examination and verification process [1]. Hence, the buyers lack the benefit of appropriate spectacle dispensing process [12]. Expert opinions and skills are very much needed in the selection of lens characteristics such as lens design, material, refractive index, coatings, measurements (i.e., monocular pupillary distance), and fitting heights. Similarly, guidance in the selection of frame material, A and B size, vertex distance, wrap, and pantoscopic angle are essential characteristics that are completely ignored when the spectacle is bought online [13]. Lack of quality check, failure in assessing the frame fit on the face, and risk of inferior or unspecified lens material are some of the additional drawbacks. The dearth of awareness of these drawbacks among the general public could be one reason why they still opt for online buying of spectacles. 

Potential risks of online buying

The quality of spectacles bought online is always in comparison with the spectacles dispensed in optometry practices [4]. Online purchase of spectacles may particularly be crucial in terms of bifocals and PALs, as these require more optimized fitting with proper measurements [12]. Risk of accidents and falls, reduced contrast sensitivity, and depth perception have been reported in elderly patients with multifocal lenses [14,15]. This increases the need for a standard and careful fitting and dispensing process [4]. Issues with optical centration distance, fitting heights, refractive correction, and lens prescription with cylindrical axis outside tolerance (prescription errors) were reported more online compared to offline purchases of eyewear [4,8]. It was also estimated that 40% of online-bought spectacle lenses failed to achieve the optical and impact-resistant American National Standards Institute (Z80.1.) [1]. In some cases, purchasing spectacles online has reported patient dissatisfaction with some defects in frame color, frame size, and poor product quality [4,8]. Using any improperly designed or fitted eyeglasses creates public health implications such as visual discomfort, reduced visual performance, high risk of falls, and decreased ocular protection against trauma [13].

Study rationale

Spectacles ordered online or purchased directly from optical stores should be verified to ensure the quality of the frame and compliance with optical tolerances set by national and international standards [4]. However, this verification process is not feasible for online purchases. While the COVID-19 pandemic has increased overall online shopping, consumer behavior toward prescription eyewear purchases needs further study [1-3]. Online purchases are often viewed by buyers as a convenient way to replace or acquire new eyewear, but they may have far-reaching effects beyond what is initially perceived [8]. The preference for purchasing spectacles online may depend on factors such as age, ease of access to online platforms, trendy collections, competitive pricing, and other considerations. Recognizing the importance of understanding consumer perspectives on purchasing prescription eyewear online in the post-COVID-19 era in India, we conducted this study. The study's findings may have implications for the eyewear industry and for individuals looking to purchase spectacles online.

## Materials and methods

A prospective cross-sectional online survey-based study design was employed, after ethical clearance was obtained from the Institutional Ethics Committee of Sri Ramachandra Institute of Higher Education and Research (reference number: CSP/21/MAY/94/327) in accordance with the Declaration of Helsinki guidelines. The study survey was conducted over a six-month period, from June 2021 to November 2021, following the second wave of COVID-19 in India [7]. The sampling method employed was non-probability purposive sampling. An online informed consent was obtained from all the participants who took part in the study. Inclusion criteria for the study were participants who had recently purchased single-vision glasses, bifocals, PALs, and prescription sunglasses through an e-commerce or online platform. Participants who had purchased Plano (without power) single-vision spectacles, sunglasses, polaroid eyewear, and near-vision readers were excluded from the study.

The online survey was administered to participants through Google Forms (Google LLC, Mountain View, California, United States). This electronic form comprised four sections (see Appendix): Section A presents the study participants with a clear and detailed explanation about the purpose of the study and a consent form to voluntarily participate in the study. Section B gathers demographic information. Section C collects details about the type of refractive error, type of purchase, online platform used, the frame style, lens type, and any lens enhancements. Section D includes a questionnaire developed and validated by Alderson et al. [4], which was used with minimal modifications after obtaining permission. This questionnaire contained questions on the appearance, fit and comfort, distance and near vision, acceptability and safety of spectacles, participants' preferences for future online or offline purchases based on their last experience, and difficulties or symptoms experienced while wearing spectacles purchased online. All questions were close-ended, except for one open-ended question that asked participants to rank their reasons for preferring or not preferring online purchases based on their personal experiences. The electronic form/invitation to participate in this study was sent to phone numbers and email IDs, and participants were also asked to share it with their contacts. The collected data were analyzed using PASW Statistics for Windows, version 18.0 (released 2009, SPSS Inc., Chicago) for descriptive and inferential statistics.

## Results

**Figure 1:**
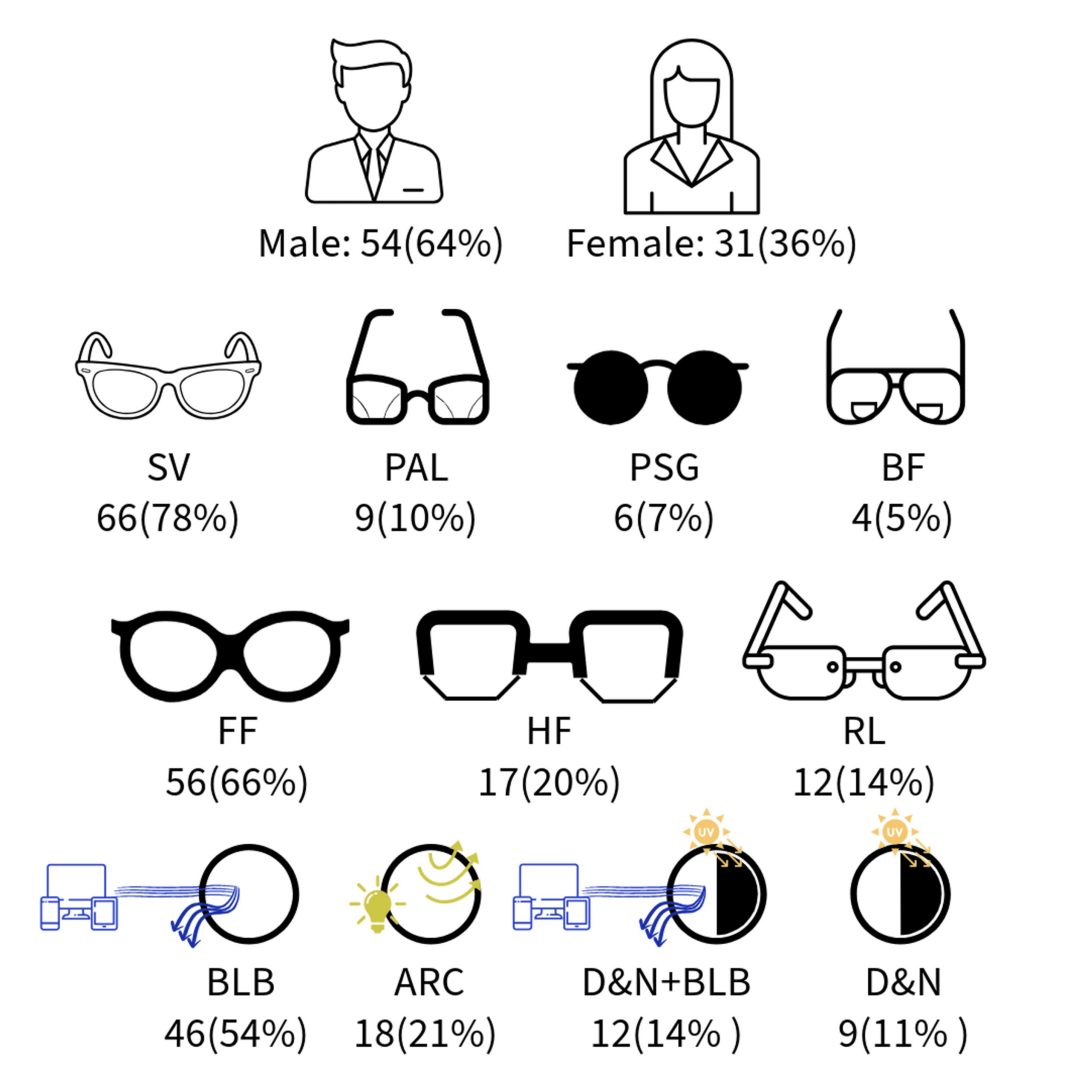
Trends observed in Indian online eyewear purchases among 85 participants SV: single vision; PAL: progressive additive lens; PSG: prescription sunglass; BF: bifocal; FF: full frame; HF: half frame; RL: rimless, BLB: blue-light blocking; ARC: anti-reflection coating; D&N: day and night This figure was designed by the corresponding author using Adobe Express.

There were a total of 85 respondents involved in the survey. The sample comprised 54 males and 31 females with a mean (standard deviation (SD)) age of 30 (9) years. Fifty-nine (69%) participants belonged to the age group of 20-30 years. Among all the participants, single-vision spectacle buyers were the most numerous (N = 66), followed by PALs (N = 9), prescription sunglass wearers (N = 6), and bifocal wearers (N = 4). Myopia (N = 55) was the most common refractive error among the study samples, followed by astigmatism (N = 10), hyperopia (N = 7), and presbyopia (N = 13). The majority of participants preferred buying full frames (N = 56), followed by half (supra) frames (N = 17), and rimless frames (N = 12). Plastic frames (N = 49) were the most preferred frame material compared to metal frames (N = 36). The most preferred lens enhancements included blue-light blocking lenses (N = 46), antireflection coating (N = 18), day-and-night lenses with blue-light blocking (N = 12), and only day-and-night lenses (N = 9). Figure 1 illustrates trends in online eyewear purchases. 

The most popular online portal for buying was Lenskart (N = 42), followed by Specsmakers (N = 25), GKB Optical (N = 7), John Jacobs Eyewear (N = 6), and Coolwinks (N = 5). Regarding the appearance of their spectacles, around 68% of the participants reported that their eyeglasses looked good, whereas 15% rated them as poor. Although 66% found the fit and comfort of the frame to be good, 29% reported bad fit and comfort, which is concerning. Regarding distance vision, 68% of the participants were happy with their vision, whereas 24% found it only to be satisfactory (Table 1). 

**Table 1:** Responses from survey questions 1-4

Questions	Good	Satisfactory	Poor
How did you find the appearance of the glasses?	58 (68%)	14 (17%)	13 (15%)
How did you find the fit and comfort of the frame?	56 (66%)	4 (5%)	25 (29%)
Did you find your distance vision through your lenses to be?	58 (68%)	20 (24%)	7 (8%)
Did you find your near vision through your lenses to be?	76 (89%)	5 (4%)	6 (7%)

Although 89% reported good near vision as many participants were wearing single-vision lenses, a few participants (three out of nine progressive wearers) reported poor near vision with the online-bought glasses. When it comes to the acceptability of online-bought spectacles based on comfort, appearance, and vision, 68% of the participants found them acceptable, while 32% felt unsafe using their glasses (Table 2). 

**Table 2:** Responses from survey questions 5 and 6

Questions	Yes	No
Did you find the glasses acceptable (please consider comfort, appearance, vision)?	58 (68%)	27 (32%)
Did you feel in any way unsafe in these glasses?	27 (32%)	58 (68%)

**Figure 2:**
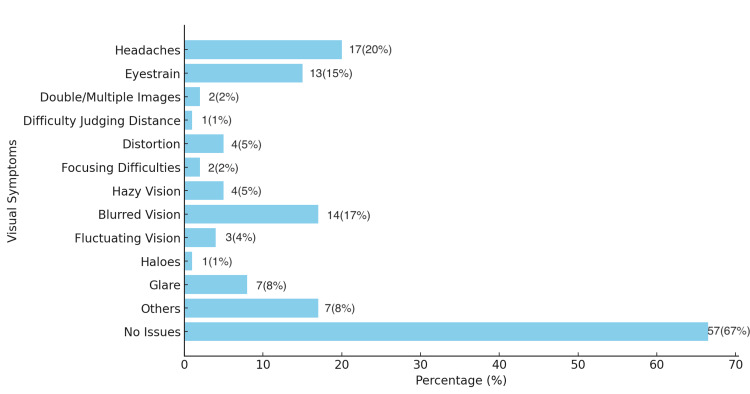
Visual symptoms experienced by wearers of glasses bought online (question 9)

This could be due to the multiple symptoms experienced by the buyers, such as headache (N = 17), blurred vision (N = 14), eye strain (N = 13), and glare (N = 7) (Figure 2).

Furthermore, 66% of the participants often preferred online eyewear purchases after COVID-19, and 60% expressed a preference for continuing to buy spectacles online after their recent experience with online-bought glasses. Meanwhile, 40% of the participants wanted to buy their next pair of glasses offline due to their unconvincing experiences with online-bought glasses. A statistical comparison between the questions "Which method of purchasing spectacles do you often prefer?" and "Based on your recent experience, where do you think you will purchase your next pair of spectacles?" showed no significant difference (p = 0.428), suggesting that the majority who have mostly preferred online buying continue to prefer online purchases.

**Table 3:** Responses from survey questions 7 and 8

Questions	Online	Offline
Which method of purchasing spectacles do you often prefer?	56 (66%)	29 (34%)
Based on your recent experience where do you think you will purchase your next pair of spectacles from?	51 (60%)	34 (40%)

The study participants also expressed their preferences and non-preferences for online purchases. The highly preferred factors were convenience, offers/discounts, and a wide range of collections, whereas the non-preferred reasons for online purchases included poor quality of spectacles, visual disturbances, and incorrect power in the glasses (Table 4).

**Table 4:** Ranking of preferences and non-preferences for online-purchased spectacles (question 10)

Rank	Reasons for preferences	Reasons for non-preferences
1	Convenience in buying	Poor quality of the spectacle
2	More offers/discounts	Visual disturbances
3	Wide range of collections	Wrong power
4	Door-step delivery	Received a different frame size/color
5	Return/Exchange	Received broken product

## Discussion

India’s eyewear market is being significantly driven by rapid urbanization and changing lifestyle trends. The COVID-19 pandemic, with restrictions like social distancing and self-isolation to minimize physical interactions, prompted many consumers to shift to e-commerce, resulting in a surge in online eyewear sales [2,3]. In addition, the increased use of digital screens and the rise of various online platforms and social media have significantly raised awareness about eye health and influenced eyewear choices [16]. The growing reach of e-commerce and online retail platforms is creating substantial opportunities for India's eyewear market. These platforms provide consumers with a convenient way to explore and buy eyewear products from the comfort of their homes [2,10,16].

To the best of our knowledge, this is the first study to report trends, satisfaction, and preferences regarding online eyewear purchases among the Indian population after COVID-19. Among the participants, 78% purchased single-vision spectacles, compared to PALs (10%), prescription sunglasses (7%), and bifocals (5%). This indicates that the younger age group, primarily aged 20-30 years (69%), showed a stronger preference for online purchases. As reported by the Ministry of Electronics and Information Technology (MeitY), India has experienced significant growth in Internet users, surpassing 500 million in recent years. This trend has been particularly prominent among younger demographics, who are increasingly seeking fashionable and affordable eyewear options [16,17].

Myopia (65%) was the most prevalent refractive error among our study single-vision lens wearers. The National Family Health Survey (NFHS) also highlights a growing trend in the increased use of digital gadgets, contributing to a rise in myopia and other eye conditions, which is driving up the demand for corrective eyewear [16]. Plastic frames (58%) were the most preferred frame material compared to metal frames (42%), highlighting buyers' preference for lighter-weight frames as a corrective eyewear material. The most sought-after lens enhancement by the buyers was blue-light blocking lenses (68%). Increased exposure to digital screens in day-to-day work is leading to a higher incidence of vision-related problems, which may explain why participants are opting for blue-light blocking lenses, marketed to relieve digital eye strain, although this remains a gray area [16,18]. Although few participants opted for prescription sunglasses for sun protection, day-and-night lenses (25%), scientifically known as photochromic lenses, were preferred due to their suitability for both outdoor and indoor activities in regular use [19]. Seventy percent of the study participants expressed satisfaction with the appearance of their glasses, as observed in previous studies [1]. Although 66% of the participants were content with the fit and comfort of their glasses, 29% were not satisfied. This suggests that selecting a frame according to the patient's facial dimensions and assessing the frame's fit both during selection and at the time of spectacle delivery are vital [13].

Factors such as age (>40 years), the design complexity of multifocal lenses, and poor near vision with online-bought PALs (33%), as observed in this study, could influence the decision to purchase PALs or bifocal lenses. A previous study has reported that participants who bought PALs online were not comfortable with their measurements and faced difficulties while reading and closer work [4]. Progressive lens users who reported poor near-visual quality in our study may have experienced this due to inaccurate measurements. It is also noted that only very few websites or e-commerce platforms allow users to enter their pupillary distance (PD) and fitting height, which are essential measurements. For PALs, major risk factors for accidents and falls in older adults have been associated with blurring in the lower visual field and distortion at the periphery of the lenses [14,15]. Therefore, it is important that PALs are dispensed only under the guidance of a qualified optometrist or optician, as these lenses require specific measurements and markings, and practitioners are trained in this area [4,13].

In our study, convenience, discounts, and a wide range of collections were factors intended to attract buyers to online spectacle purchases, which aligns with previously reported literature [10,17]. However, on the downside, poor spectacle quality, multiple visual disturbances, and incorrect prescription power of the ordered glasses were identified as major drawbacks. A study that evaluated the optical quality, comfort, and user preferences for spectacles purchased online in the UK concluded that participants were generally more comfortable buying spectacles from optometry practices rather than online. A greater number of online spectacles were found to be unsafe or unacceptable due to poor frame fit, cosmetic appearance, and inaccurate optical centration [4]. Another study, which assessed the prescription spectacles ordered via the Internet reported that several spectacles were provided incorrectly, with almost 45% failing in at least one parameter of optical or impact testing. They concluded that nearly half of the prescription spectacles delivered by online vendors did not meet the optical requirements for patients' visual needs or the physical requirements for patient safety [1]. As observed in our study, 32% of the respondents reported that their glasses were unsafe or unacceptable in terms of comfort, appearance, and vision. In addition, 33% of the participants experienced multiple visual symptoms, such as headache (20%), blurred vision (17%), and eye strain (15%), which is concerning. These issues may be attributed to incorrect prescriptions, inaccurate optical centration, or prismatic effects in the spectacles [1,4,13]. 

To summarize, this study explored various factors influencing the online purchase of spectacles in India after COVID-19. While there has been a surge in online eyewear purchases since its onset, it is crucial to understand the associated pros and cons, which have been highlighted in our study. However, the study was limited by its relatively small sample size and the use of a purposive sampling technique, which may affect the generalizability of the findings to a broader population. Furthermore, no direct testing or assessment of the participants' online-purchased spectacles was conducted, which could have provided a more objective evaluation of quality and safety. Nonetheless, the insights gathered here form a strong foundation for future research, offering valuable directions for more extensive studies involving larger, more diverse sample populations and incorporating direct assessments of online-purchased spectacles in the current era. Future studies could also explore the impact of technological advancements such as virtual try-on features and augmented reality tools on consumer behavior and decision-making when purchasing spectacles online.

## Conclusions

This study highlights trends and satisfaction levels with online-purchased spectacles among the Indian population after COVID-19. While the study primarily involves younger age group consumers who mostly use single-vision lenses and generally find online purchases satisfactory, concerns remain regarding poor frame fit, comfort, and safety. Given the link between online spectacles and adverse effects like eye strain and headaches, offline purchases with standard verification protocols may offer better visual outcomes.

## Appendices

Study questionnaire

Section A 

Survey on Trends and Satisfaction in Buying Powered Spectacles Online

Purpose: We are conducting a survey to understand the trends and customer satisfaction related to purchasing powered spectacles or eyewear online. Your input will help us analyze consumer experiences and improve services in the eyewear industry.

Eligibility: You are eligible to participate if you have purchased powered spectacles or eyewear online within the past 6 months.

Voluntary Participation: Participation in this survey is entirely voluntary. You may withdraw at any time without any consequences.

Confidentiality: Your responses will remain anonymous and will be used solely for research purposes. No identifying information will be shared or published.

Duration: The survey will take approximately 5-10 minutes to complete.

E-Consent: By clicking "Agree" and proceeding to the survey, you confirm that you have read and understood the purpose and criteria of the study, meet the eligibility criteria and consent to participate in this survey voluntarily.

Section B 

Name:

Age:

Sex:

Occupation:

Phone no. (optional):

Section C 

Type of refractive error (power):

Myopia (short-sightedness) 

Hyperopia (long-sightedness) 

Astigmatism (cylindrical power)

Presbyopia (40 years and above)

Other, specify _______________

Type of purchase: 

Online 

Offline 

Online platform (website/application) used for spectacle purchase: ______________

Spectacle purchased date: _____________

Frame style purchased:

Full-rim frame

Half-rim frame (Supra)

Rimless frame 

Other, specify _______________

Frame material purchased: 

Metal frame 

Plastic frame 

Other, specify _______________

Lens type purchased: 

Single-vision lens 

Bifocal lens 

Progressive lens 

Sunglass/tinted lens (with power)

Other, specify _______________

Lens enhancements (additional lens benefits opted) 

Anti-reflection coating 

Blue-light cut/blocking 

Photochromic (day-and-night lens)

Other, specify _______________

Section D

1) How did you find the appearance of the glasses?

a) Good

b) Satisfactory

c) Poor

2) How did you find the fit and comfort of the frame?

a) Good

b) Satisfactory

c) Poor

3) Did you find your distance vision through your lenses to be?

a) Good

b) Satisfactory

c) Poor

4) Did you find your near vision through your lenses to be?

a) Good

b) Satisfactory

c) Poor

5) Did you find the glasses acceptable (please consider comfort, appearance, vision)?

a) Yes

b) No

6) Did you feel in any way unsafe in these glasses?

a) Yes

b) No

7) Which method of purchasing spectacles you often prefer?

a) Online

b) Offline

8) Based on your recent experience where do you think you will purchase your next pair of spectacles from?

a) Online

b) Offline

9) Did you experience any of these difficulties or symptoms when wearing the spectacles? (Can choose multiple symptoms)

a) Headaches

b) Eyestrain

c) Double/multiple images

d) Difficulty judging distance

e) Distortion

f) Focusing difficulties

g) Hazy vision

h) Blurred vision

i) Fluctuating vision

j) Haloes

k) Glare

l) Others

m) No issues

10) What are your reasons for preferring or not preferring online purchases of spectacles? (Please rank your preferred and non-preferred reasons in order of 1, 2, 3, etc. based on your personal experience.)
